# A single institutional study on survival and fertility outcomes in malignant ovarian germ cell tumour patients

**DOI:** 10.3332/ecancer.2025.2002

**Published:** 2025-09-30

**Authors:** Pambinkavil Sivaraman Raji, Anitha Thomas, Vinotha Thomas, Anjana Joel, Dhanya Susan Thomas, Rachel G Chandy, Ajit Sebastian, Abraham Peedicayil

**Affiliations:** 1 Department of Gynaecologic Oncology, Christian Medical College, Vellore 632004, Tamil Nadu, India; 2 Department of Medical Oncology, Christian Medical College, Vellore 632004, Tamil Nadu, India

**Keywords:** ovarian germ cell tumour, fertility sparing surgery, reproductive outcome, survival outcome

## Abstract

**Introduction:**

Malignant ovarian germ cell tumours (MOGCTs) are rare and account for 70% of malignant ovarian tumours in adolescents. Hence, fertility preservation is an important issue among this population. In this study, we aim to analyze our experience of MOGCT treatment with special emphasis on outcomes of fertility-preserving treatment and on survival outcomes.

**Materials and methodology:**

All patients with MOGCT who underwent treatment in our institution, from January 2010 to December 2018, were included and the clinicopathologic characteristics, chemotherapy details, recurrence characteristics and follow-up data were analysed. The patients who underwent fertility-sparing surgery were contacted over the telephone to collect reproductive and menstrual outcome details.

**Results:**

A total of 84 patients were included in the study of which upfront surgery was followed by adjuvant chemotherapy in 44 (52.4%) patients and neoadjuvant chemotherapy was followed by interval debulking surgery and adjuvant chemotherapy in 8 patients. Fertility-sparing surgery was performed in 49 (58.3%) patients and 17 patients attempted conception and 13 (76.5%) succeeded in spontaneous pregnancy, resulting in 11 live births. All of them resumed their menstrual cycle within 1 year of treatment. With the median follow-up of 46 months, the mean 3-year disease-free survival and overall survival of 84 patients with MOGCT were 86.9% and 96.4%, respectively.

**Conclusion:**

Fertility-preserving surgery with appropriate adjuvant treatment has excellent survival and fertility outcomes among patients with early as well as advanced-stage disease. Hence, it is a safe and effective option for young females with MOGCTs.

## Introduction

Malignant ovarian germ cell tumour (MOGCT) is a rare entity. It is derived from primordial germ cells of the ovary and it accounts for 5% to 7% of all ovarian malignancies [1].

The different histological types are dysgerminoma, yolk sac tumour, immature teratoma, chorio carcinoma, embryonal carcinoma, poly embryoma and mixed MOGCT.

These tumours are commonly seen in the adolescent and young adult age group, with peak incidence at 15–19 years of age. It accounts for 70% of malignant ovarian tumours in this age group [2, 3]. Hence, fertility preservation is an important issue among this population. Over the last 30 years, the management of ovarian MOGCT has changed dramatically after the introduction of fertility-sparing surgery and chemotherapy [4]. According to the recent evidence, the standard treatment for MOGCT is fertility-sparing surgery irrespective of the cancer stage when fertility preservation is desired, along with comprehensive surgical staging with or without adjuvant chemotherapy [5, 6]. The standard chemotherapy regimen is Bleomycin, Etoposide and Cisplatin (BEP), and ovarian germinal epithelium usually gets affected by the antiproliferative effects of chemotherapeutic agents. Although it can result in ovarian dysfunction or failure, many women can still achieve resumption of menstrual cycle and successful pregnancies [4]. Very few studies from India have evaluated the oncological and reproductive outcomes of MOGCT [7, 8]. We conducted a retrospective chart review among all the MOGCT patients treated between 2010 and 2018, mainly focusing on the fertility outcome.

## Materials and methods

This is a retrospective study done in the Department of Gynecologic Oncology in a tertiary care centre. All patients with MOGCT who underwent treatment in our institution, between January 2010 and December 2018 were included. This also includes those patients who were referred to our centre soon after surgery for further treatment. Histopathological slide review was done by an expert gynecologic pathologist at our institution. The following data were collected from electronic medical records: age at diagnosis, parity, Eastern cooperative oncology group (ECOG) performance status, types of surgery, The International Federation of Gynecology and Obstetrics (FIGO) stage, residual disease after surgery, tumour classification and grade, chemotherapy details, disease recurrence and follow up details such as resumption of menstruation, fertility and successful pregnancies. Patients who had fertility-sparing treatment were contacted over the phone to obtain the menstrual and reproductive details. Fertility sparing surgery (FSS) is defined as the preservation of the uterus, one tube and one ovary. Comprehensive or complete surgical staging includes peritoneal washings, peritoneal biopsies, omentectomy and retroperitoneal lymph node assessment.

Reproductive outcomes include resumption of menstrual cycles after the treatment, number of pregnancies and live births. The pregnancy rate and live birth rate were calculated by dividing the number of successful pregnancies by the number of patients who tried to conceive and number of successful deliveries by number of patients who tried to conceive, respectively.

Progression-free survival (PFS) was calculated as the time interval in months between the date of last treatment (surgery or chemotherapy) and the date of recurrence or death. Overall survival (OS) was calculated as the time interval in months, between the date of last treatment (surgery or chemotherapy) and the date of last follow up or death.

Univariate and multivariate analysis were done to identify the factors associated with reproductive and survival outcomes.

*p*-value <0.05 was regarded as statistically significant. SPSS software, version 21.0, was used for statistical analysis.

## Results

A total of 84 patients were included. The patient characteristics are presented in Table 1. Mean age at diagnosis was 23.7 ± 6.8 years (range 12 to 42) and 52 patients (61.9%) were nulliparous. The common histological types encountered were 27 (32.1%) patients with dysgerminoma, immature teratoma in 26 (31%), yolk sac tumours in 12 (14.3%) and mixed germ cell tumours in 19 (22.6%) patients. FIGO stage was I in 44 (53.7%), II in 2 (2.4%), III in 34 (41.5%) and IV in 2 (2.4%) of patients. The majority of patients 73 (87%) had regular menstrual cycles before treatment and one patient had not attained menarche. One patient had primary amenorrhea at presentation. The majority of the patients, 72 (85.7%), presented with abdominal pain and mass per abdomen; one patient who presented with primary amenorrhea was later diagnosed as XY pure gonadal dysgenesis.

The mean hemoglobin level was 10.6 ± 1.6 g/dL (range 6.4 to 15) and the mean body mass index (BMI) was 21 ± 3.6 kg/m^2^ (range 13.3–30.7). ECOG performance status was assessed in all the patients. The majority of the patients 78 (85%) had ECOG ≤1 and one patient had ECOG 3, who presented as an advanced stage of dysgerminoma and did not undergo surgery. Out of 84 patients, 2 patients received only chemotherapy. Surgery was performed in the remaining 82 patients with or without chemotherapy.

The operative and histopathological details are presented in Table 2.

The mode of surgery was laparotomy in all patients except one, who had a laparoscopic hysterectomy. The majority of the patients, 43 (52.4%), underwent unilateral salpingo oophorectomy (USO) and staging. Nineteen patients underwent a hysterectomy with the removal of both ovaries for the following reasons: Fourteen patients were parous and did not want to preserve fertility. Among the five nulliparous patients, two had tumour infiltration into adjacent structures, including the uterus and bladder. One was a 45-year-old nulliparous woman who did not want fertility preservation, and another had previously undergone surgery for a mixed germ cell tumour at an outside center. The last patient opted for complete surgery even after counselling due to logistical reasons.

Complete/optimal surgical staging, which includes peritoneal staging and lymph node evaluation, was performed in 79 (96.3%) patients. Residual tumour defined as any visible tumour left after surgery, was present in three patients, of which one had Figo stage IV B yolk sac tumour with lung metastases who underwent USO alone and received adjuvant chemotherapy and later developed pelvic recurrence. The other patient had stage IIIC malignant mixed GCT with inoperable disease for which an omental biopsy alone was taken. The third patient had stage III dysgerminoma with a nodal mass of 2 cm, which was left behind as it was adherent to major vessels.

Chemotherapy (Neoadjuvant/adjuvant) was received by 63 (75%) out of 84 patients. The details of chemotherapy are summarised in Table 3. The most common chemotherapeutic regimen used was BEP in 38 (60.3.%) out of 63 patients, EP (etoposide and cisplatin) in 12 (19%) patients, JEB (bleomycin, etoposide and carboplatin) in 2 (3.1%) patients, VAC (vincristine, doxorubicin and cyclophosphamide) in 2 (3.1%) patients and vinblastin, Ifosfomide and cisplatin (VbIP) in 2 (3.1%) patients.

Three patients received carboplatin and paclitaxel combination regimen. A total of 13 patients with stage 1 ovarian germ cell tumours were managed with post operative surveillance without requiring chemotherapy. The histological types were as follows: eight cases of immature tertoma, three cases of dysgerminoma, one case of yolk sac tumour and one case of malignant mixed germ cell tumour. Evidence supports surveillance in stage I dysgerminoma and stage I, grade 1 immature teratoma. For yolk sac and mixed germ cell tumour, our decision was individualised as they came after FSS done at a peripheral center and came many months later for follow up. Imaging and tumour markers were normal and advised to follow up closely. There was one patient who became unfit for chemotherapy. She was diagnosed with a mixed germ cell tumour that presented in an advanced stage. There were five patients who defaulted on adjuvant chemotherapy due to personal reasons. The mean number of chemotherapy cycles was 3 (range 1–9).

One patient presented with stage IVB yolk sac tumour with brain and lung metastases. She received whole-brain radiotherapy and salvage chemotherapy using EP.

The reproductive and obstetric outcome data are summarised in Table 4.

FSS was performed in 49 (58.3%) patients, but reproductive and obstetric outcome data were available in 45 patients. Of the 49 patients who underwent fertility preservation surgery, 45 (91.8%) patients resumed their menstrual cycle. All of them resumed their menstrual cycle within 1 year of treatment. The remaining four patients who did not resume periods included a Turner mosaic patient diagnosed on karyotyping and one with premature ovarian failure. The other two patients were lost to follow up.

Among the 49 patients who underwent FSS, 30 had stage I disease, 1 had stage II and 18 had stage III. In total, there were 34 cases of stage III disease, of which 18 (52.9%) underwent FSS. The remaining 16 patients underwent complete surgical staging, including the removal of the uterus and ovaries. The reasons for this included multiparity and no future fertility requirements in 12 patients. Among the remaining four nulliparous patients, two underwent complete surgery due to large tumours infiltrating adjacent structures, one opted for complete surgery preoperatively and one had bilateral ovarian tumours. Of the 49 patients who underwent FSS, 17 patients attempted conception and 13 (76.5%) patients succeeded in pregnancy. All of them conceived spontaneously except one, who underwent ovulation induction. Of the remaining four patients, two patients were under treatment for infertility with ovulation induction during the study period. One patient with premature ovarian failure and another one with male factor infertility were advised *in vitro* fertilisation. Both of them were not willing for the same. Four patients who got married were not trying for pregnancy at present.

Fifteen patients were still unmarried post treatment, during the study period. Four patients had married already and had got children prior to the treatment and not attempted conception post treatment.

Nine patients were lost to follow up. There were 15 pregnancies from 13 patients, resulting in 11 live babies. This includes four patients with stage III disease who had successful pregnancies. There was one first-trimester miscarriage and one neonatal death with a renal anomaly. Two were pregnant during the study period.

The pregnancy rate was 88.2% (15/17) and the live birth rate was 64.7% (11/17). Of the 13 patients, 8 patients (61.5%) received adjuvant chemotherapy and 6 (46.2%) received GnRH agonist (inj.

Leuprolide acetate monthly once for 3 to 4 doses as a part of ovarian function preservation) during chemotherapy. The mean time interval between completion of treatment and pregnancy is 3.9 ± 2.3 years (range 1 to 9 years).

In univariate analysis, age of the patient, Figo stage of the disease and use of adjuvant chemotherapy were found to be not related to resumption of menstrual cycles post treatment. However, it was found that the use of GnRH agonist is significantly associated with resumption of menstrual cycles post treatment (*p* value - 0.004). Multivariate analysis could not be done due to the small sample size.

To know the factors related to positive pregnancy outcome, univariate analysis was done for the age of the patient, Figo stage of the disease, histopathology, presence of residual disease, use of adjuvant chemotherapy and use of GnRH analogues. None of the factors was found to be related to the same.

**Figure 1.**
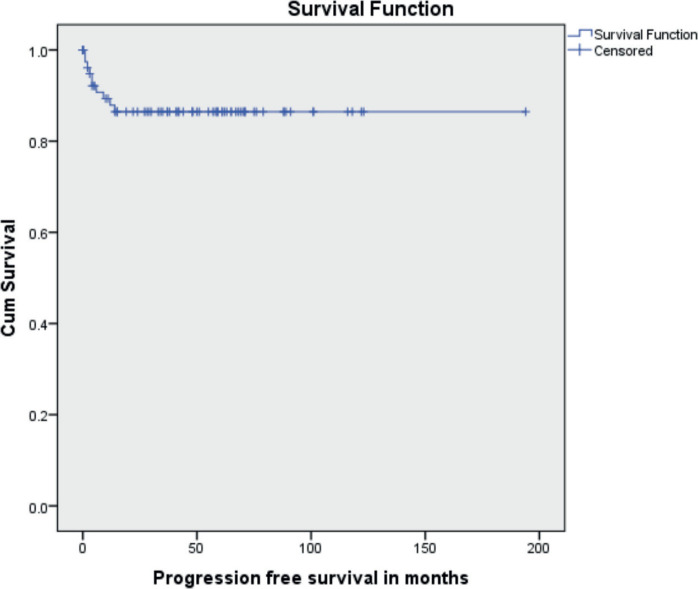
Kaplan–Meir survival curve showing PFS.

**Figure 2.**
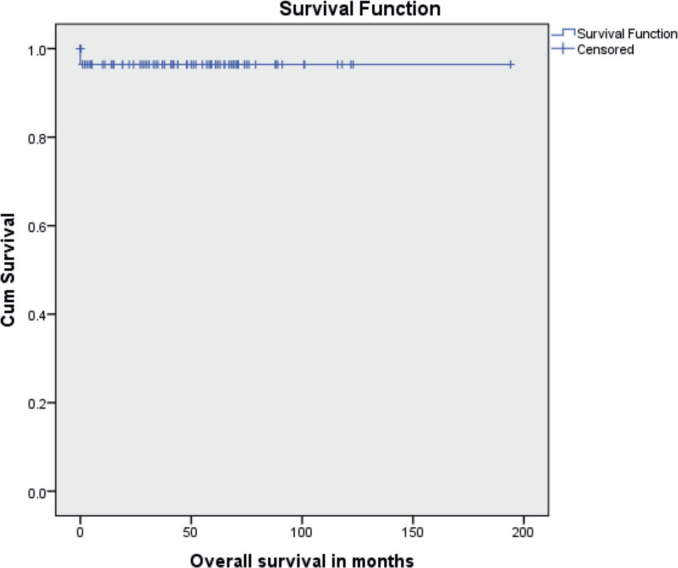
Kaplan-Meir survival curve showing OS.

The median follow-up time was 46 months (range 1 to 194 months). Ten patients (11.9%) had recurrent disease and three patients (3.6%) died due to the disease. Among the ten patients who developed recurrence, four were under post-operative surveillance when the recurrence was diagnosed. The 5-year disease-free survival (DFS) was 88.1% and the 5-year OS was 96.4% (Figures 1 and 2). In the univariate analysis, age of the patient, performance status, Figo stage of the disease, histopathology, preoperative elevated tumour markers and adjuvant chemotherapy were not associated with reduced DFS. However, incomplete surgical staging was significantly associated with reduced DFS (*p* value 0.002) on univariate analysis.

**Figure 3.**
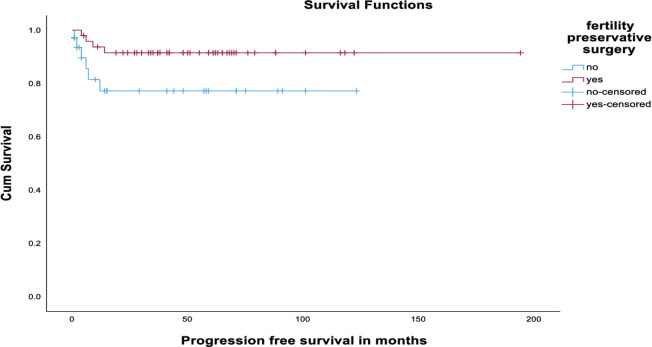
Kaplan-Meir survival curve showing DFS among fertility-sparing and non-fertility-sparing surgery groups.

**Figure 4.**
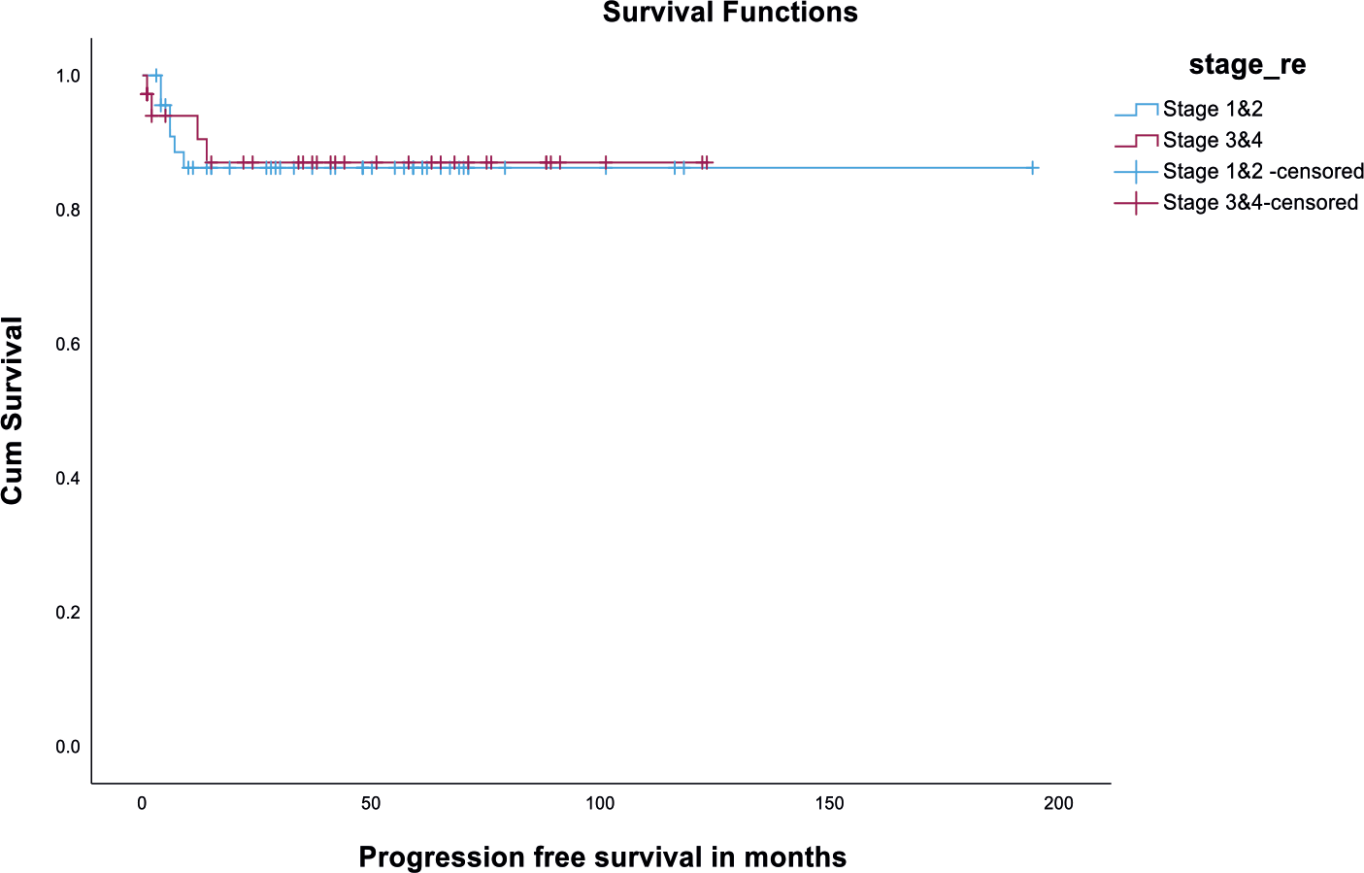
Kaplan Meir curve showing DFS among early versus advanced stage.

There was no significant difference in DFS among the fertility-preserved surgery group and fertility non-preserving surgery group (*p* value - 0.21) (Figure 3). Also, there was no significant difference in DFS among early (stages I and II) versus advanced disease (Stages III and IV). It is depicted in Figure 4. Disease recurrence details are presented in Table 5.

The majority of patients had disease recurrence in pelvis (8/10), and one had metastases in the liver along with pelvic disease and other one had retroperitoneal node metastases.

One patient with stage IVB yolk sac tumour and another one with stage III malignant mixed GCT were advised second-line chemotherapy for recurrence, but defaulted and were unable to be contacted. The remaining eight patients were alive at the time of follow up.

Three patients died of the disease. One was a young girl who presented in the advanced stage, dysgerminoma. She was started on single-agent carboplatin in view of poor performance status. She developed renal failure and expired after 2 weeks of chemotherapy.

The second patient had a malignant mixed germ cell tumour and received 2 cycles of BEP chemotherapy. She presented with loose stools and multiple seizure episodes after 1 week of the last chemotherapy to the emergency department and expired on the same day with the probable diagnosis of septic shock.

The last one was again a case of malignant mixed GCT, stage III, underwent interval debulking surgery and had progression of disease after the 4th cycle of adjuvant chemotherapy. She developed febrile neutropenia along with multiple abscesses and cellulitis and expired due to septic shock.

## Discussion

Radical, non-conservative surgery was the standard treatment for any ovarian malignancy until the last three decades. Their elevated chemosensitivity, frequent unilaterality and the young age of patients have strongly supported conservative surgery as the standard approach, which is often followed by adjuvant platinum-based chemotherapy [9]. Our experience with MOGCT over the past one decade has shown promising reproductive and survival outcomes after fertility-sparing surgery with appropriate chemotherapy.

The chemotherapeutic agents used in the treatment of MOGCT and their antineoplastic effects were extensively studied. Impaired follicular maturation, cortical fibrosis and reduction in follicles are the histological changes in the ovary seen in the patients receiving chemotherapy [10]. These effects are related to the type of drug used, schedule, drug dosage and duration of treatment.

Ovarian failure is most commonly caused by alkylating agents. But short-term usage of antimetabolites, Vinka alkaloids and antitumour antibiotics is associated with reduced risk of ovarian function suppression [11]. To reduce the toxicity in children JEB (carboplatin, etoposide and bleomycin) has replaced the BEP regimen [12].

Our study has shown that 91.8% (45/49) of patients resumed their menstrual cycle within 1 year post treatment. Among the 45 patients, 31 received adjuvant chemotherapy, of which 13 patients were on ovarian function suppression using Leuprolide acetate. Although there are retrospective and prospective studies that have shown the benefit of hormonal suppression during chemotherapy to prevent gonadal dysfunction [13], no prospective studies have confirmed these findings.

However, our results suggest that hormonal suppression during chemotherapy may have a protective effect on ovarian function. Our results are similar to other reports of excellent rates of return of menstrual function (85 to 95%) after platinum-based chemotherapy for MOGCTs [14].

Brewer *et al* [15] reported that after FSS and BEP chemotherapy 71% of patients maintained their normal cycles during and after treatment, remaining patients resumed menstruation within 6 months of completing chemotherapy and 22% of patients became pregnant.

Zanetta *et al* [16] investigated 138 patients who underwent FSS, of whom 58% received adjuvant chemotherapy. All but one post menarchial patient resumed menstrual function post treatment with a median time of 5 months. They reported that 27.4% of patients treated with adjuvant chemotherapy and 21.8% of patients treated only with FSS attempted to conceive, with a pregnancy rate of 80% and 100%, respectively [16].

Low *et al* [17] found that 91.5% of 47 patients with MOGCTs had resumed their normal menstrual function after chemotherapy completion. There were 14 live births in the chemotherapy group.

Tamauchi *et al* [18] reported reproductive outcome in 105 patients who received FSS with a median follow up period of 10.4 years and found that 42 of 45 patients who desired childbirth became pregnant with a total of 65 pregnancies and 56 babies.

Tangir *et al* [19] studied reproductive outcome in 64 patients with a median follow up of 122 months and found a 76% pregnancy rate in 38 patients who attempted conception post treatment. Talukdar *et al* [7] recently reported that a total of 18 of 21 patients who received BEP chemotherapy and underwent FSS, resumed menstruation and 10 of them delivered 13 healthy babies.

Gershenson *et al* [4] have reported that of the 71 patients who remained disease-free after completion of treatment, 62 (87%) patients resumed menstrual cycles and 24 (34%) of them reported having 37 healthy babies.

Dellino *et al* [20], reported their study of 24 patients who underwent FSS, of which 5 patients attempted conception and succeeded spontaneously.

Zamani *et al* [21] in 2021 studied 72 patients who underwent FSS, of which 60 (83%) patients recovered regular menstruation post treatment and 78% of patients in the adjuvant treatment group resumed menstruation and 19 of the 26 patients (73%) who attempted conception proceeded to delivery without any infertility treatment.

In our study, the pregnancy rate was 88.2% and live birth rate was 64.7%. Out of 13 patients who conceived after FSS, 8 patients (61.5%) received adjuvant chemotherapy, which shows that reproductive outcomes are promising after FSS, even in patients who received adjuvant chemotherapy. Similar results have been shown by Weinberg *et al* [22] with a pregnancy rate of 75% and a live birth rate of 65%.

In our center, the OS of 84 patients with MOGCT was 96.4% (81/84) and the DFS was 88.4% (74/84), even though 38 (45.2%) patients had disease spread beyond ovaries at the time of diagnosis. These data are similar to other case series by Tangir *et al* [19] which reported approximately 100% survival among patients with early-stage disease and upto 75% in advanced-stage disease]. When we compared DFS among early (stages I and II) versus advanced stage (stages III and IV), it was 100% versus 97%, respectively, with no statistical significance (Figure 4).

However, in the fertility-preserved patients, the OS was 100% and DFS was 91.8%. Of the 49 patients who underwent fertility-sparing surgery (USO alone or USO plus staging), 4 patients developed recurrence, whereas 6 among 33 patients who underwent TAH BSO ± staging developed recurrence. This supports the fact that fertility-sparing surgery does not have a worse outcome with respect to survival or recurrence. These data were supported by an earlier study by Kurman and Norris [23], who reported no worse prognosis in 182 patients with MOGCT with disease confined to one ovary who underwent fertility-sparing surgery.

We found that incomplete surgical staging was significantly associated with reduced DFS (*p* value 0.002) on univariate analysis. But there was no significant difference in DFS among the fertility-preserved surgery group and fertility non-preserving surgery group (*p* value - 0.21).

Therefore, fertility-sparing surgery is preferred when a woman wishes to preserve her reproductive function and there is no gross evidence of disease spread to the contralateral ovary, even though there is extraovarian disease.

Our study has shown that the potential for pregnancy after FSS and chemotherapy appears to be unaffected by chemotherapy. Of the 17 patients who attempted conception,13 succeeded in the pregnancy, of which 8 of them had received chemotherapy. Except for one patient who had ovulation induction prior to pregnancy, rest all conceived spontaneously with a pregnancy rate of 88.2% resulting in 11 full term live births. Previous studies have reported fertility rates from 60 up to 95% [13–17]. Hence, our results support that the reproductive capacity is unaffected by FSS and chemotherapy. We also found that the risk of recurrence and survival does not seem to be affected by the type of surgery performed (conservative versus radical).

Therefore, young females with MOGCT can be reassured of their reproductive potential and excellent survival after conservative surgery and chemotherapy.

Our study is a real-world series of patients with MOGCTs treated in a single referral institute that shows oncological and reproductive outcomes. It is among a few Indian studies evaluating the survival and fertility outcomes of patients with MOGCT. The limitations of our study are those associated with any retrospective analysis: small sample size and incomplete follow-up details. Additionally, the present study did not include pediatric MOGCT patients.

## Conclusion

Fertility-sparing surgery with or without adjuvant chemotherapy has been shown to have excellent survival outcomes in young women with MOGCTs; hence, it should be considered as an option while counselling. Good menstrual and fertility outcomes are achievable even after fertility-sparing surgery and chemotherapy, even in advanced-stage disease. Long-term follow up should be emphasised for the patients after treatment.

## Conflicts of interest

The authors declare that they have no known competing financial interests or personal relationships that could have appeared to influence the work reported in this paper.

## Funding

No funding was received for this study.

## Consent to participate

As it is a retrospective study consent was waived off.

## Consent for publication

NA.

## Ethical approval

Christian Medical College Vellore, Institutional Review Board approved (IRB number 13691/2020).

## Author contributions

**Raji PS:** Writing – review and editing, Writing – original draft, Formal analysis, Data curation, Conceptualisation. **Anitha Thomas:** Writing – review and editing, Resources, Data curation. **Rachel George Chandy:** Writing – review and editing, Supervision, Methodology, Conceptualisation. **Dhanya Susan Thomas:** Writing – review and editing, Visualisation, Resources. **Vinotha Thomas:** Writing – review and editing, Supervision, Resources, Formal analysis, Conceptualisation. **Abraham Peedicayil:** Writing – review and editing, Supervision, Resources, Methodology, Formal analysis, Conceptualisation. **Ajit Sebastian:** Writing – review and editing, Writing – original draft, Supervision, Resources, Formal analysis, Data curation, Conceptualisation.

## Availability of data and material

The data will be made available on request.

## Code availability

NA.

## Figures and Tables

**Table 1.** Baseline patient characteristics.

Variable	*N* = 84
Age (years)	23.7 ± 6.8 (12–42)
BMI (kg/m^2^)	21 ± 3.6
Parity Nulliparous Parous	52 (61.9) 32 (38.1)
Presenting symptoms Abdominal pain Mass per abdomen Distension AUB Primary amenorrhoea Primary infertility Hemoperitoneum	42 (50.0) 16 (19.04) 16 (19.04) 02 (2.3) 02 (2.3) 02 (2.3) 04 (4.76)
ECOG performance status 0 1 2 3	21 (25) 57 (67.9) 05 (6) 01 (1.1)
Hemoglobin (g/dL)	10.6 ± 1.6
Preoperative tumor markers hCG Normal (≤5 mIU/mL) Elevated (>5 mIU/mL) Not tested LDH Normal (105–333 IU/L) Elevated (>333 IU/L) Not tested AFP Normal (≤40 mcg/L) Elevated (>40 mcg/L) Not tested CA-125 Normal (0–35 U/mL) Not elevated Not tested	48 (57.1) 27 (32.1) 09 (10.7) 13 (15.5) 57 (67.8) 14 (16.7) 40 (47.6) 41 (48.8) 3 (3.6) 24 (28.6) 39 (46.4) 21 (25)

Values expressed as *n* (%) or mean ± SD and/or range

ECOG, Eastern Co-operative Oncology Group; AUB, Abnormal uterine bleeding; hCG, human chorionic gonadotropin; LDH, Lactate dehydrogenase; AFP, Alpha Fetoprotein; CA 125, Cancer antigen

**Table 2.** Operative and histopathological details.

Variable	*N* = 82
Type of surgery USO alone USO + staging TAH + BSO TAH + BSO + staging TAH + USO + staging BSO + staging TLH + USO Excision of the ovarian Mass alone (suboptimal) Ileocecal mass excision Along with omentectomy	06 (7.3) 43 (52.4) 04 (4.9) 15 (18.3) 06 (7.3) 04 (4.9) 01 (1.2) 02 (2.4) 01 (1.2)
FIGO staging Stage I Stage II Stage III Stage IV	44 (53.7) 02 (2.4) 34 (41.5) 02 (2.4)
Completeness of resection R0/R1 R2	79 (96.3) 03 (3.7)
Histopathology Dysgerminoma Immature teratoma Yolk sac tumor Mixed germ cell tumour	27 (32.1) 26 (31) 12 (14.3) 19 (22.6)

Values expressed as *n* (%)

USO, Unilateral salpingo-oophorectomy; BSO, Bilateral salpingo-oophorectomy; TAH, Total abdominal hysterectomy; TLH, Total laparoscopic hysterectomy; FIGO, International Federation of Gynecology and Obstetrics

**Table 3.** Chemotherapy details.

Setting & regimen	*N* = 65
**NACT alone + Surgery**	*n* = 11
BEP	10
BEP + **EP**	01
**Upfront** s**urgery + Adjuvant chemotherapy**	*n* = 44
BEP	27
EP	12
Carboplatin + Paclitaxel	02
BEP + EP	02
JEB	01
**Interval debulking surgery + Adjuvant chemotherapy**	*n* = 8
BEP	01
BEP, EP	02
JEB, **VAC**	01
EP, **VbIP**	01
BEP, **EP and VbIP**	01
EP, Carboplatin and Paclitaxel	01
BEP,** VAC**	01
**Chemotherapy alone**	*n* = 2
BEP	01
Carboplatin	01
**Patients who did not require chemotherapy**	13
**Patients who defaulted adjuvant chemotherapy**	05
**Unfit for chemotherapy**	01

Values expressed as ***n***

**NACT**, Neoadjuvant chemotherapy; **BEP**, Bleomycin, etoposide** and cisplatin; EP**, etoposide and cisplatin; **JEB**, bleomycin, etoposide** and carboplatin; VAC**, vincristine, doxorubicin** and cyclophosphamide; VbI**P, vinblastin, Ifosfomide** and cisplatin**

**Table 4.** Reproductive and obstetric outcomes.

Outcome	Value
Total number of FSS	49/84 (58.3)
Post treatment return of menstrual function	45/49 (91.8)
Mean time to return of menstrual function	8 months
Post treatment attempt to conception	17/49 (34.7)
Number of patients who got conceived	13/17 (76.4)
Number of patients who received both surgery and chemotherapy	8/13 (61.5)
Spontaneous conception	12
Infertility treatment (Ovulation induction)	1
Live births	11
Miscarriage	1
Renal anomaly and neonatal death	1
Total number of pregnancies*	15
Pregnancy rate	15/17 (88.2)
Live birth rate	11/17 (64.7)

Values expressed as *n* (%)

* includes two patients who became pregnant for the second time after miscarriage and anomaly

**Table 5.** Pattern of recurrence and its treatment.

Variable	Value
Median (range) follow up (months)	46 (1–194)
Total number of recurrences	10
Site of recurrence Pelvis Liver and pelvis Retroperitoneal nodes	08 01 01
Primary histopathology Yolk sac tumor Immature teratoma Dysgerminoma Mixed germ cell tumor	01 04 03 02
Treatment of recurrence Second line chemotherapy Secondary debulking surgery Defaulted treatment	05 04 01

Values expressed as *n*
